# Coupling of Size Exclusion Chromatography to High Throughput Charge Detection Mass Spectrometry for the Analysis of Large Proteins and Virus-like Particles

**DOI:** 10.1021/acs.analchem.4c06084

**Published:** 2025-01-29

**Authors:** Raj A. Parikh, Lohra M. Miller, Benjamin E. Draper, Lavelay Kizekai, Balasubrahmanyam Addepalli, Michelle Chen, Matthew A. Lauber, Martin F. Jarrold

**Affiliations:** 1 Chemistry Department, Indiana University, Bloomington, Indiana 47405, United States; 2 Megadalton Solutions Inc., 3750 E. Bluebird Lane, Bloomington, Indiana 47401, United States; 3 Waters Technology Corporation, 34 Maple Street, Milford, Massachusetts 01757, United States; 4 Waters Technology Corporation, 6330 Hollister Avenue, Goleta, California 93117, United States

## Abstract

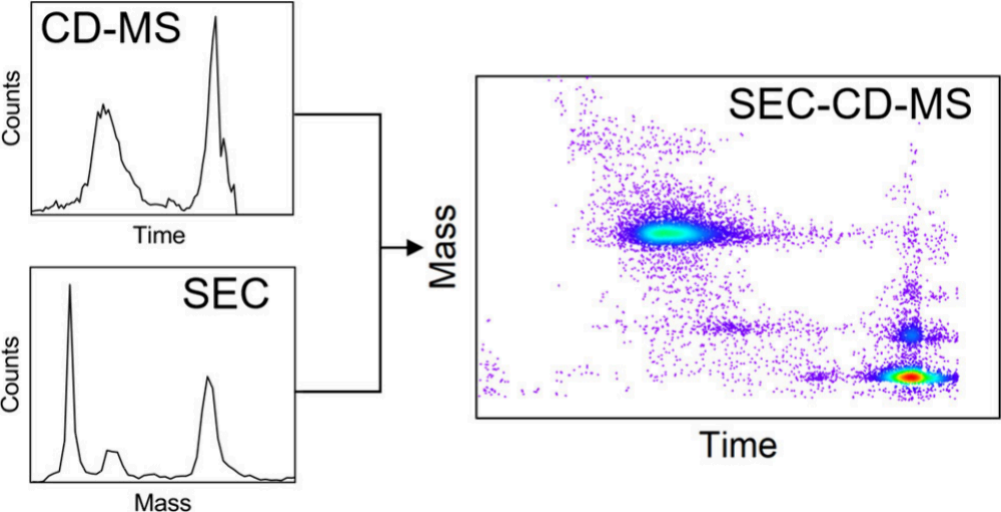

Charge detection mass spectrometry (CD-MS) is an emerging single-particle technique where both the *m*/*z* and charge are measured individually to determine each ion’s mass. It is particularly well-suited for analyzing high mass and heterogeneous samples. With conventional MS, the loss of charge state resolution with high mass samples has hindered the direct coupling of MS to separation techniques like size exclusion chromatography (SEC) and forced the use of lower resolution detectors. Here, we show how CD-MS, leveraging high-throughput methods such as multiple ion charge extraction (MICE), can match the time scale of SEC, extending the samples amenable to separation analysis by SEC-CD-MS into the megadalton regime and beyond. As part of this work, we have developed low flow ultrawidepore (1000 Å pore size) SEC using narrow bore columns to optimize the coupling between SEC and CD-MS. The analysis of monoclonal antibodies, thyroglobulin, bacteriophage Qβ virus-like particles (VLPs), and hepatitis B virus VLPs, showcases the capabilities of SEC-CD-MS over a broad mass range including the high mass range previously inaccessible for online separation with MS. These findings are complemented by a parallel study using multiangle light scattering (SEC-MALS). SEC-CD-MS and SEC-MALS provide complementary information that is valuable for characterization of complex biologics and nanoparticles. Finally, our results open the door to integration of high throughput CD-MS with other separation techniques for both large and small macromolecules.

## Introduction

Mass spectrometry is an essential tool for the characterization of biomolecules. When coupled to a chromatography method such as size exclusion chromatography (SEC),^1^ the hyphenated analytical workflow can be used to identify by mass, analytes separated by size. To date, most SEC-MS workflows have used conventional MS techniques that are best suited to small macromolecules (<200 kDa) where charge state resolution can be achieved. To accurately measure the size of larger protein complexes, virus-like particles (VLPs), and gene therapy vectors, chromatography workflows have traditionally relied on light-based techniques. These techniques combine tunable ultraviolet absorption (TUVs), refractive index (RI), and multiangle light scattering (MALS) detectors to calculate the molar mass of the analyte.^2^

With charge detection mass spectrometry (CD-MS), the limitation of conventional MS to small macromolecules (due to the loss of charge state resolution) was overcome by directly measuring the masses of individual ions. The ions travel through a detection cylinder and the signals from the induced charge are used to determine the *m*/*z* ratio and charge for each ion. Using CD-MS, the upper mass range available to MS has been extended into the gigadalton regime.^3−5^ The first coupling of SEC to CD-MS was achieved by Viode and co-workers who used single pass CD-MS to measure mass distributions for large polymers.^6^ In single pass CD-MS the ions pass through the detection cylinder once. This implementation has the advantage of high throughput, but with only a single measurement for each ion, single pass CD-MS has a high charge limit of detection and low resolution (due to the low precision of each *m*/*z* and charge measurement).^7,8^ Much lower limits of detection and much higher resolution can be achieved by embedding the detection cylinder in an electrostatic linear ion trap and signal averaging.^9,10^ Using this approach, the limit of detection has been lowered to ions with a single charge,^11^ and the charge can be measured with a precision of better than 0.2 e (elementary charges).^12,13^ Off-line CD-MS measurements offer high resolution and high sensitivity for intact biomolecules that are beyond the upper mass limit of conventional MS. However, there is a penalty for the dramatic improvements in the limit of detection and resolution–the measurement time. The time to measure 5000 individual ions is a useful benchmark for the measurement time. Until recently, it typically took 30–60 min to record a CD-MS spectrum containing 5000 individual ion measurements, a time scale that is incompatible with chromatographic separations. Matching the time scales is the main barrier to direct coupling of chromatography to CD-MS.

Individual ion measurements on the Orbitrap platform provide an alternative way of measuring the mass distributions for heterogeneous samples.^14,15^ However, Orbitrap DMT (Direct Mass Technology) requires a much longer trapping time than CD-MS and cannot measure the charge as accurately, so the time to measure a spectrum is longer as well.^16,17^ Bones and co-workers reported efforts aimed at coupling SEC to Orbitrap DMT.^18^ However, the results were not very encouraging with only a few ions detected from the eluting peaks. Furthermore, the instrument settings had to be manually adjusted for each eluting peak which requires prior knowledge of the species present. Some more recent results reported by Heck and co-workers appear to offer a higher throughput.^19^ They used SEC-Orbitrap DMT to study the degradation of an immunoglobulin M antibody. However, they increased the throughput by reducing the trapping time, which degrades the accuracy of the charge measurement, and lowers the mass resolution. The potential of SEC-Orbitrap DMT has not yet been realized because of constraints on the ion throughput owing to the long trapping time needed for accurate charge measurements for individual ions by Orbitrap DMT. Furthermore, the measurements performed so far have been limited to smaller macromolecules that are easily within the range of conventional MS. Thus, the high mass capabilities of single ion MS measurements have not yet been exploited.

Both CD-MS and Orbitrap DMT are slower than conventional MS because each ion is analyzed individually. In both approaches it is possible to simultaneously trap more than one ion and track each ion’s signal to determine its charge and *m*/*z*.^20^ However, there is an upper limit to the number of ions that can be trapped simultaneously. The signals from the oscillating ions are analyzed by fast Fourier transforms (FFTs). The oscillation frequency is used to determine the *m*/*z* and the FFT magnitude is proportional to the charge. If two ions have similar oscillation frequencies their peaks overlap in the FFT frequency space, and their measured charges become unreliable. The recently developed MICE (Multiple Ion Charge Extraction) algorithm overcomes this problem^21^ and allows CD-MS measurements to be performed at up to 200 ions/sec, in its most recent incarnation. This allows a CD-MS spectrum containing 5000 ions to be measured in well under 1 min. In addition to this increase in capacity, pulsed mode CD-MS has increased the sensitivity of CD-MS by up to 2 orders of magnitude and facilitates multiple ion trapping.^22^

Biologics, nanoparticles, and other high molar mass samples often have complex formulation buffers that include surfactants, free protein, and oligonucleotides. The analyte of interest may be present at a low concentration relative to other components in solution. This presents a challenge for single particle methods because most of the measurement time is spent detecting and processing signals for species that are irrelevant, leading to long measurement times to obtain the required signal for the analyte of interest. Online separation and desalting with SEC will allow for a fast, sensitive, and automated analysis of the species of interest by CD-MS. In addition, aggregation is an important concern for many biologics and as we show below, SEC-CD-MS allows solution-based aggregates to be distinguished from electrospray artifacts, allowing SEC-CD-MS to provide more accurate characterization data.

To optimize the coupling between SEC and CD-MS, we have developed low flow ultrawidepore (1000 Å pore size) SEC using narrow bore columns, in contrast to the wide bore SEC columns usually used to analyze high molar mass samples.^23,24^ Indeed, smaller pore size particles have been packed and commercialized in the form of narrow bore columns. However, these narrow-bore, ultrawidepore (1000 Å pore size) SEC columns are novel in their ability to expand fractionation range and resolving power to increasingly larger analytes. The output from the low flow 1000 Å SEC column is split, with a minor component electrosprayed in an off-axis configuration. The resulting ions are analyzed by high throughput CD-MS. The performance of SEC-CD-MS was assessed using two proteins and two VLPs with molar masses ranging from 150 kDa to over 4 MDa. Using the MICE algorithm, the CD-MS measurements are fast enough to collect enough ions during the peak elution time for analyte characterization, placing SEC-CD-MS firmly in the bioprocessing analytical toolbox. Finally, the SEC-CD-MS measurements for the VLPs were compared with SEC-MALS results for the same samples. The comparison highlights the complementary nature of the two techniques for large macromolecule characterization.

## Experimental Methods

### Materials and Samples

See Supporting Information.

### Analytical Scale and Narrow Bore SEC-UV

UV chromatograms for the four samples were collected to compare the relative separation efficiency of 4.6 versus 2.1 mm ID SEC columns. These columns were prepared using 3 μm 1000 Å SEC particles (GTxResolve HO-PROSEC 3 μm 1000 Å particles) packed into h-HST hardware (Waters Corporation, Milford, MA).^25^ An Arc Premier System was configured with two mobile phase systems: one comprised of 20 mM phosphate, 276 mM NaCl, 5.4 mM KCl pH 7.4 (2× dPBS) and another comprised of a volatile composition consisting of 200 mM ammonium acetate (99%) and 0.1% formic acid (1%) prepared in 18.2 MΩ water. The column temperature was maintained at 30 °C and flow rates of 0.4 and 0.083 mL/min were applied to each 4.6 and 2.1 mm ID column, respectively. Samples of 5.0 μL 5 mg/mL bovine thyroglobulin, 10.0 μL 5 mg/mL NISTmAb, 20.0 μL 2 mg/mL Qβ VLPs, and 5.0 μL 0.67 mg/mL HBV VLPs with residual CP149 dimer were injected onto the 4.6 mm ID column. Injection volumes for 2.1 mm ID column chromatography were 1.0, 2.1, 4.2, and 1.0 μL, respectively. Eluting analytes were detected by UV absorbance at 280 and 260 nm.

### Online SEC-CD-MS

Native SEC separations were performed on a Waters ACQUITY UPLC M-Class System equipped with a 2.1 mm × 300 mm GTxResolve Premier SEC 1000 Å 3 μm Column and a 5 μL sample loop. The output from the SEC column was connected to the electrospray source using 360 μm OD x 50 μm ID fused silica tubing (Polymicro Technologies). The flow rates and mobile phase for all separations can be found in Table S1 (in Supporting Information). Following column elution, the flow at the electrospray emitter was reduced to 5 μL/min by splitting the flow with a MicroCross PEEK fitting (Idex P-889). A portion of the split flow went to the electrospray source with the balance being discarded. The remaining arm of the PEEK cross was used for a high voltage electrical connection for electrospray emitter. The electrospray emitter was a 15 μm diameter fused silica PicoTip Emitter (New Objective, FS360-75-15-D-5). To minimize contamination of the CD-MS instrument, the potential to the electrospray emitter was turned off prior to the elution of the SEC void peak and elution of any salts introduced by sample injection. The sample concentrations were 1 mg/mL for NISTmAb, thyroglobulin, and Qβ VLPs, and 0.67 mg/mL for HBV VLPs with residual CP149 dimer.

Measurements were performed on a prototype Megadalton CD-MS instrument. Briefly, ions enter the instrument through a metal capillary and are thermalized in the FUNPET, an electrospray interface optimized for transmission of a wide range of masses.^26^ Upon exiting the FUNPET, ions travel through an RF-only hexapole and a segmented RF-only quadrupole. Ions that exit the quadrupole are focused into an energy analyzer that selects a band of ion kinetic energies centered on 130 eV/z. The transmitted ions are focused into an electrostatic linear ion trap (ELIT) that consists of two end-caps that act as ion mirrors, with the detection cylinder embedded between them.^27^ Ions were measured in continuous trapping mode with 104.6 ms trapping events that give a charge RMSD of ∼0.6 e. All trapping events were timestamped and matched to the SEC injection to generate chromatograms. The MICE algorithm enabled ion fluxes up to 200 ions/s to be analyzed.^21^

### SEC-UV-MALS-RI for Molar Mass and Other Biophysical Attributes

MALS measurements were performed with an Arc Premier System consisting of a r-QSM (Quaternary Solvent Manager) with 100 μL Mixer, 2489 Detector (Analytical MaxPeak Premier Flow Cell, 10 mm, 500 nL), r-FTN-SM (Flow Through Needle-Solvent Manager) with 15 μL MaxPeak Premier Needle and 100 μL MaxPeak Premier Extension Loop, CH-30A heater with a MaxPeak Premier Active Preheater 18.5” and postcolumn tubing to TUV made of a 0.005” ID × 22.5” LG MP35N Welded Tube. The instrument was equipped with three sequential detectors: a TUV detector, an 18-angle DAWN MALS Detector with online Quasi-Elastic Light Scattering (QELS) (Waters I Wyatt Technology, Goleta, CA), and Optilab Refractive Index (RI) Detector (Waters I Wyatt Technology). The mobile phase used in this analysis was 20 mM phosphate, 276 mM NaCl, and 5.4 mM KCl in water, pH 7.4 (2× strength dPBS). The SEC-MALS system constants were established using BSA at 2 mg/mL. 20 μL of 2 mg/mL Qβ and 14.4 μL of 0.67 mg/mL HBV VLPs with residual CP149 dimer were injected onto a GTxResolve Premier SEC 3 μm 1000 Å 7.8 × 300 mm Column. A typical protein dn/dc (specific refractive index increment) value of 0.185 mL/mg was used for both Qβ and HBV CP149.

## Results and Discussion

### Low Flow Size Exclusion with a Volatile Mobile Phase

Recently, narrow bore (2.1 mm) SEC have been employed as a means to reduce sample consumption and enhance mass spectrometry sensitivity and compatibility.^23^ We have continued this effort to facilitate the coupling of large analyte size separations with direct online mass measurements. Our miniaturization of SEC began with the selection of recently developed 3 μm 1000 Å silica particles that feature a hydroxy-terminated poly(ethylene oxide) bonding protected with bridged ethylene surface cross-linking.^28^ Analytical (4.6 × 300 mm) and narrow bore (2.1 × 300 mm) columns were prepared with these particles using low adsorption hardware, and optimal packing of the resulting particle beds was confirmed by uracil plate counts of 73 and 46K, respectively (albeit on a test instrument optimized for 4.6 mm ID columns). Uracil is a small molecule analyte used in these procedures to measure the theoretical plate count of the column and thus the overall quality and physical properties of the packed bed.

**Figure 1:**
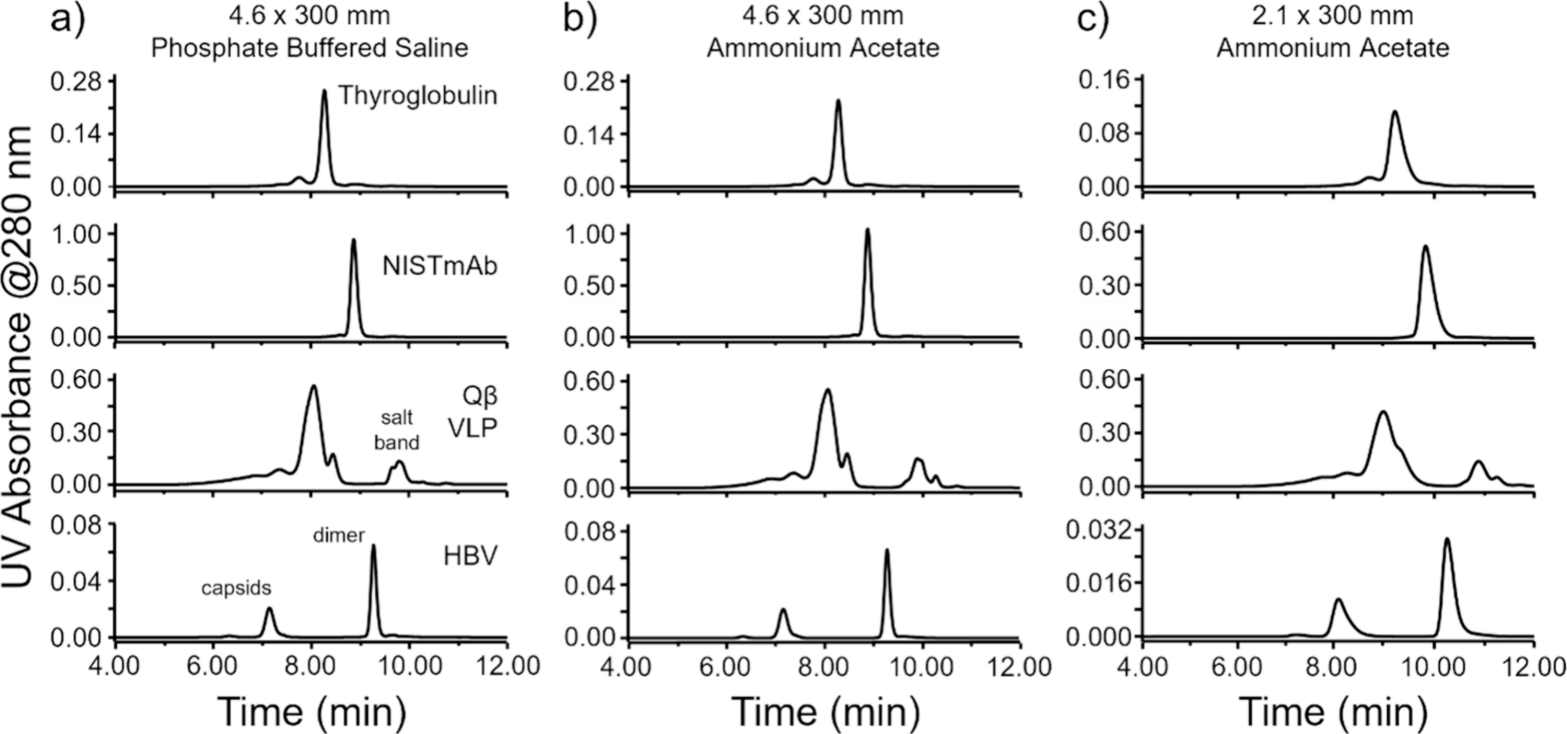
UV SEC chromatograms to assess the miniaturization of widepore SEC columns. (a) Chromatograms obtained with a 4.6 mm × 300 mm, 1000 Å, 3 μm column with 2× concentration dPBS mobile phase versus (b) chromatograms obtained with a 4.6 mm × 300 mm, 1000 Å, 3 μm column using a volatile mobile phase of 200 mM ammonium acetate/0.1% formic acid mixed in the ratio of 99:1. (c) Injection and flow rate scaled chromatograms collected with the volatile mobile phase system on a 2.1 mm × 300 mm column.

The resolving power of these columns was first compared using traditional SEC-UV applied to the two protein and two VLP samples: NISTmAb, bovine thyroglobulin, Qβ VLP, and HBV Cp149 VLP. Analytical bore (4.6 mm ID) separations were acquired with both a 2x strength dPBS and volatile ammonium acetate-based mobile phase. Figure 1 presents these chromatograms in panels a) and b). Consistent peak profiles and recoveries were observed for all 4 samples despite the change from a traditional phosphate buffer mobile phase to an MS-compatible ammonium acetate solution, which confirms there are minimal secondary interactions in these newly designed columns. That is, elution times changed by no more than 1% and peak areas changed by no more than 12%. For both method conditions, an average, effective peak capacity was estimated using the elution time window corresponding to the first and last eluting species detected across all samples and the average peak widths observed at 50% height for each main sample component. An effective peak capacity of 14.7 was calculated for the 4.6 mm ID column with the PBS based mobile phase; a near equivalent value of 14.6 was calculated for the ammonium acetate runs. Because of the complexity of these samples, it should be noted that these values underestimate the true resolving power. For instance, the Qβ VLP sample has four species that elute at slightly different times in its main peak (see below). Despite this caveat about the peak width measurements, this comparison remains useful for assessing separation performance.

Using the same UHPLC instrument, we subsequently tested a narrow bore (2.1 mm ID) column with the mobile phase flow rate appropriately scaled for the diameter of the column. Comparable profiles were achieved, although the effective peak capacity was reduced to 8.6 (see Figure 1c). This ∼40% reduction is consistent with previously reported results and the effects of pre- and postcolumn dispersion effects on SEC columns with smaller volumes.^23,29^

With the miniaturization of wide pore SEC established through SEC-UV measurements, the next issue to address was configuring the 2.1 × 300 mm column on a microflow LC instrument (Waters ACQUITY UPLC M-Class System) and then coupling the effluent flow to the electrospray source of the CD-MS instrument. As described in the Experimental Methods section, it was necessary to split the flow from the SEC column to reduce the flow to the electrospray source. After splitting, the flow to the electrospray emitter was around 5 μL/min. The emitter was orientated orthogonally to the metal capillary where ions enter the CD-MS instrument, and the capillary sampled smaller droplets at the edge of the electrospray plume to optimize desolvation and desalting of the ions.

### SEC CD-MS of NISTmAb and Thyroglobulin

**Figure 2:**
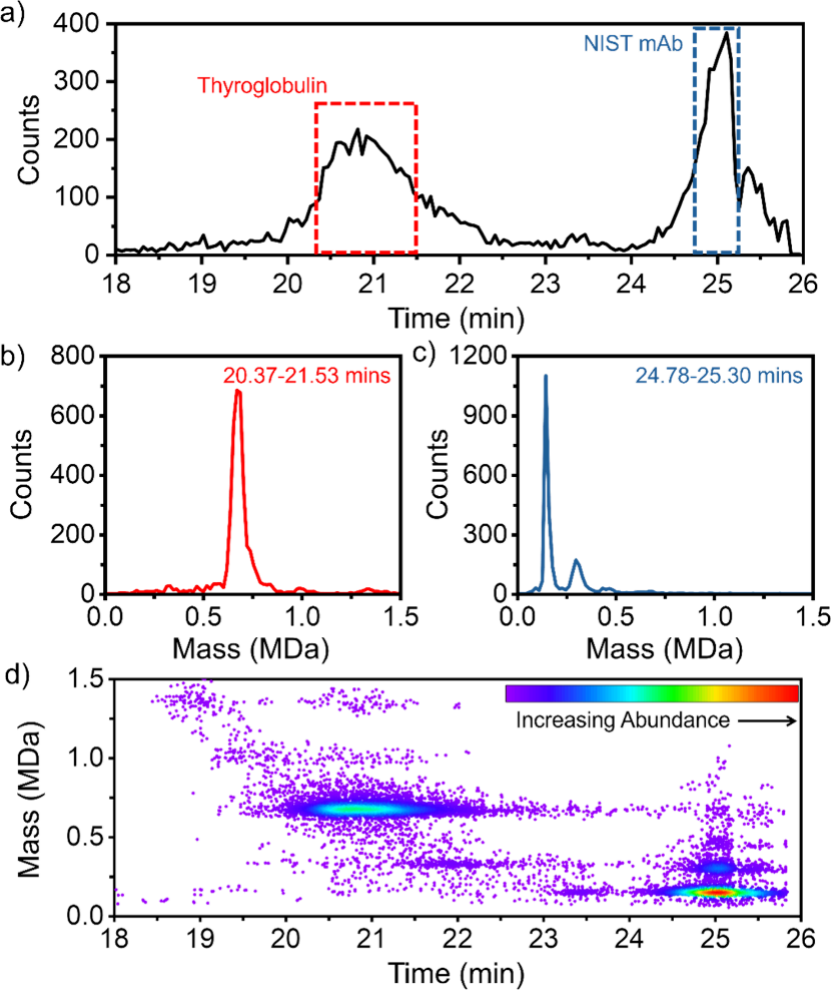
SEC-CD-MS analysis of thyroglobulin and NISTmAb. (a) Total ion count chromatogram. The bin size is 3 s. (b, c) Mass distributions for the first and second peaks in the total ion count chromatogram. The dashed red and blue lines in (a) delineate the ions included in the mass histograms. The bin size is 17 kDa. (d) 2D abundance map of mass versus time for the thyroglobulin and NISTmAb separation.

Thyroglobulin, a protein precursor of thyroid hormones, presents as a homodimeric glycoprotein with a 660 kDa molar mass.^30^ For an initial test of SEC-CD-MS, a solution containing thyroglobulin and NISTmAb (∼148 kDa)^31^ was separated and measured. Figure 2a shows the time versus intensity plot for peaks eluting between 18 and 26 min. The first peak to elute (red dashed box) at 20.95 min is the larger species, thyroglobulin. The second peak (blue dashed box) is the smaller, mAb at 25.04 min. Ions within one standard deviation from the center of the first peak from 20.37 to 21.53 min (indicated by the red dashed box) were selected and their mass distribution plotted in Figure 2b. The mass distribution shows a sharp peak at 675.5 kDa which corresponds to the expected mass for thyroglobulin. The full width at half-maximum (fwhm) of the peak for the dimeric glycoprotein is 63 kDa which is within the expected range when potential glycoforms of the protein are considered.^32^ The mass distribution for the second peak in the chromatogram from 24.78 to 25.30 min (indicated by the blue dashed box) is shown in Figure 2c. The main peak in the mass distribution is at 148.5 kDa which corresponds to the expected mass for the NISTmAb monomer. There are smaller peaks in the mass distribution at 2 and 3 times the monomer mass which are probably due to multimers.

The thyroglobulin spectrum in Figure 2b was generated from 3886 single ion measurements (collected in 70 s) and the NISTmAb spectrum in Figure 2c was generated from 2781 ions (collected in 31 s). The chromatogram in Figure 2a uses 3 s binning. Thus, the number of counts on the vertical axis can be converted into an ions/s scale by dividing by 3 s. The collection rate at the maximum of the thyroglobulin peak is thus around 70 ions/s and for the NISTmAb the maximum is around 140 ions/s. Both are below the 200 ions/s maximum, beyond which the relative intensities in the spectrum can be significantly influenced by frequency overlap and ion–ion interactions.^21^ Since the mass distributions in Figure 2b and c contain a single dominant peak, the number of ion signals collected is more than enough to determine the mass accurately and faster elution times could be used.

Figure 2d shows a 2D abundance map of the SEC-CD-MS separation with mass along the vertical axis and elution time along the horizontal. The advantage of this presentation is that the lower abundance species become more visible. Before the elution of the main thyroglobulin peak, there is intensity in the 2D map in Figure 2d at a mass of 1.36 MDa and an elution time of 19 min. The mass of 1.36 MDa can be correlated with a small peak in the mass distribution in Figure 2b. It aligns well with the expected mass of the thyroglobulin tetramer (i.e., a dimer of thyroglobulin dimers). The fact that it occurs at an earlier elution time than the thyroglobulin dimer (indicating a larger hydrodynamic radius) is consistent with this assignment. Furthermore, because the retention times of the thyroglobulin tetramer are well separated from the retention times of the dimer, the half-life for dissociation of the tetramer into dimer must be comparable to or longer than the retention time. In other words, the equilibrium between the tetramer and dimer is slow. If the half-lives for dissociation and reassociation of the tetramer are significantly shorter than the retention time, the retention times of the dimer and tetramer would be the same. There is a second region of intensity in the 2D map with the same mass as the thyroglobulin tetramer but with an elution time which is similar to the elution time of the thyroglobulin dimer (20.95 min). This feature could be due to another dimer where there is a rapid equilibrium between dissociation and reassociation (i.e., a more weakly bound dimer) or it could be due to an electrospray artifact that results because some of the electrospray droplets contain multiple copies of the thyroglobulin dimer that coalesce during the droplet drying process. Between the mass of the thyroglobulin dimer and tetramer there is another band of intensity that correlates with the small peak in the mass distribution in Figure 2b at 1.01 MDa. The mass of this feature is in good agreement with the expected mass of the thyroglobulin trimer. The intensity for this peak is spread out over a broad range of elution times.

Multimers are present in the mass distribution for NISTmAb with peaks at 2 and 3 times the nominal mass (see Figure 2c). However, inspection of the 2D map in Figure 2d shows that the NISTmAb multimers have the same elution time as the monomer, suggesting that these result from either short-lived solution aggregates or electrospray artifacts. There is intensity at an elution time of 22 min that could be due to long-lived NISTmAb dimer present in solution. However, CD-MS reveals that this feature is centered on the mass of the thyroglobulin monomer (338 kDa) rather than the mass of the NISTmAb dimer (297 kDa).

The results presented above show that a 2D map of the SEC-CD-MS single particle results is valuable in helping to assign minor components present in the sample. In particular, it can help to identify long-lived (i.e., strongly bound) solution aggregates which have a different retention time than the unaggregated species, and distinguish them from short-lived (i.e., weakly bound) solution aggregates and aggregates from electrospray-induced coalescence during the droplet drying process, both of which are expected to have the same retention time as the unaggregated species.

The SEC separation performed here serves as a preliminary test of both the widepore column as well as the coupling between SEC and CD-MS instrumentation. The resolution of thyroglobulin and NISTmAb was easily accomplished with the flow parameters employed and so larger molecules were tested to explore the limitations of the column and the SEC-CD-MS technique.

### SEC CD-MS of Qβ VLPs

Bacteriophage Qβ VLPs have recently shown promise in vaccine development and as a drug delivery vehicle.^33−35^ The native bacteriophage assembles into an icosahedral *T* = 3 capsid with 180 capsid proteins (CPs).^36,37^ When overexpressed, the Qβ major capsid protein assembles into a variety of morphologies. In addition to the canonical *T* = 3 particle, an oblate geometry with 150 CPs, small and large prolate forms with 132 and 210 CPs, and a *T* = 1 particle with 60 CPs, have been observed by cryo-electron microscopy.^38,38^ Five peaks were detected In a previous CD-MS study of Qβ VLPs.^21^

**Figure 3:**
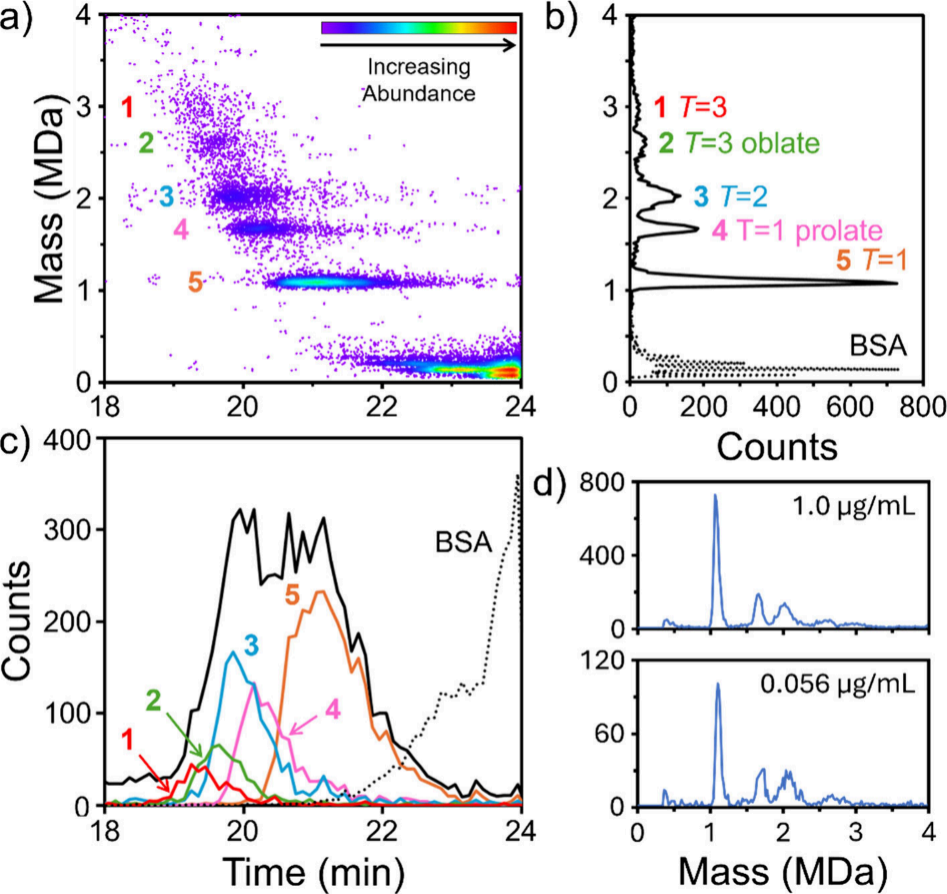
SEC-CD-MS measurements for bacteriophage Qβ VLPs. BSA was included to mark the end of the fractionation range. (a) 2D abundance map of the SEC-CD-MS separation of Qβ and BSA sample components. An abundance scale is given in the plot. (b) Mass distribution measured for the eluting sample. The ions attributed to Qβ are plotted with a solid line (>800 kDa, 20 kDa bins), and ions attributed to BSA are plotted with a dotted line (<800 kDa, 6 kDa bins). The five peaks attributed to Qβ are numbered 1 through 5 along with their tentative assignments. (c) Total ion count chromatogram for Qβ and BSA. The solid black line shows ions attributed to Qβ and the dotted line shows ions attributed to BSA. The bin size is 6 s, and the counts for BSA have been scaled down by a factor of 2.5. The colored lines show counts for the five Qβ peaks in the mass distribution. (d) Dilution study for bacteriophage Qβ VLP. Mass distributions recorded for concentrations of 1.0 and 0.056 mg/mL.

Figure 3a shows a representative 2D abundance map of the SEC-CD-MS separation of Qβ and BSA (bovine serum albumin). BSA was added to the Qβ sample to provide a marker to indicate the end of the Qβ elution so that the electrospray source could be turned off to prevent the salt band from contaminating the CD-MS instrument. Figure 3b on the right shows the mass distribution measured during elution of the Qβ and BSA sample components. The five peaks attributed to Qβ VLPs align with the peaks observed in the previous CD-MS study.^21^ However, the assignment of these peaks to Qβ geometries is not straightforward because an undefined quantity of RNA is packaged during assembly and its presence shifts the positions of the peaks. The measured masses and tentative assignments are given in Table S2 (in Supporting Information) and the tentative assignments are shown in Figure 3b.

The peaks attributed to BSA in Figure 3b and c are shown with dotted lines. The BSA intensity at elution times less than 23.5 min results mainly from stable solution phase dimers, trimers, and tetramers. There is also dimer, trimer, and tetramer that have the same retention time as the monomer. These result from labile aggregates and/or electrospray-induced coalescence. An expanded view of the 2D abundance map and mass distribution for BSA are given in Figure S1 (in Supporting Information).

The five Qβ VLP peaks in the mass distribution are not resolved chromatographically (solid black line in Figure 3c). However, the CD-MS mass resolution can be leveraged to separate ions in the mass peaks and plot their elution times. The colored lines in Figure 3c show the elution time distributions for the five Qβ VLP mass peaks in the mass distribution. The results show that the five components are only partially separated in this microflow SEC experiment. The five species (numbered 1–5 in Figure 3) elute at 19.4, 19.7, 20.0, 20.4, and 21.15 min, respectively. Multimers of BSA start to elute at around 21.25 min and the BSA monomer appears at 23.4 min in the chromatogram. The spectrum was generated with data from 15,603 ions collected in a single SEC run. A total ion chromatogram that extends to shorter elution times than Figure 3c is shown in Figure S2a. This chromatogram shows a significant tail to shorter elution times that is attributed to aggregates. The mass distribution of the ions with elution times between 13 and 19 min is shown in Figure S2b. The aggregates account for around 5% of the ions in the chromatogram.

Note that the thyroglobulin dimer (Figure 2d) elutes earlier than the Qβ *T* = 1 species (Figure 3a) while having a mass that is close to half as large (0.675 MDa versus 1.09 MDa). Figure S3 shows a 2D abundance map of the SEC-CD-MS separation of a Qβ VLPs and thyroglobulin mixture showing the shorter elution time of the thyroglobulin dimer. The SEC elution time is expected to correlate with the hydrodynamic radius,^39^ so this observation suggests that the hydrodynamic radius of the thyroglobulin dimer is larger than the radius of the Qβ *T* = 1 species, despite its much smaller mass.

To further evaluate the performance of SEC-CD-MS, a dilution study was performed for Qβ. Samples containing an initial concentration of 1 mg/mL Qβ were serially diluted down to 0.056 mg/mL Qβ. The results for the highest and lowest concentration are shown in Figure 3d, the results for all concentrations are shown in Figure S4 (in Supporting Information). The relative abundances of the main components in the mass distribution are unchanged upon serial dilution. This is a consequence of the MICE algorithm which corrects for discrimination against high intensity signals at high measurement rates. Even after a 16-fold dilution the number of ions detected is still enough to determine the masses of the main features in the mass distribution. For low concentration samples pulsed mode CD-MS could be used to increase the signal intensity by up to 100x, lowering the limit of detection by the same amount.^22^ With pulsed mode CD-MS, ions are accumulated in the hexapole and subsequently pulsed into the ELIT when the entrance end-cap is in transmission mode and the ELIT is ready to accept ions.

### SEC CD-MS of Hepatitis B Virus VLPs

**Figure 4:**
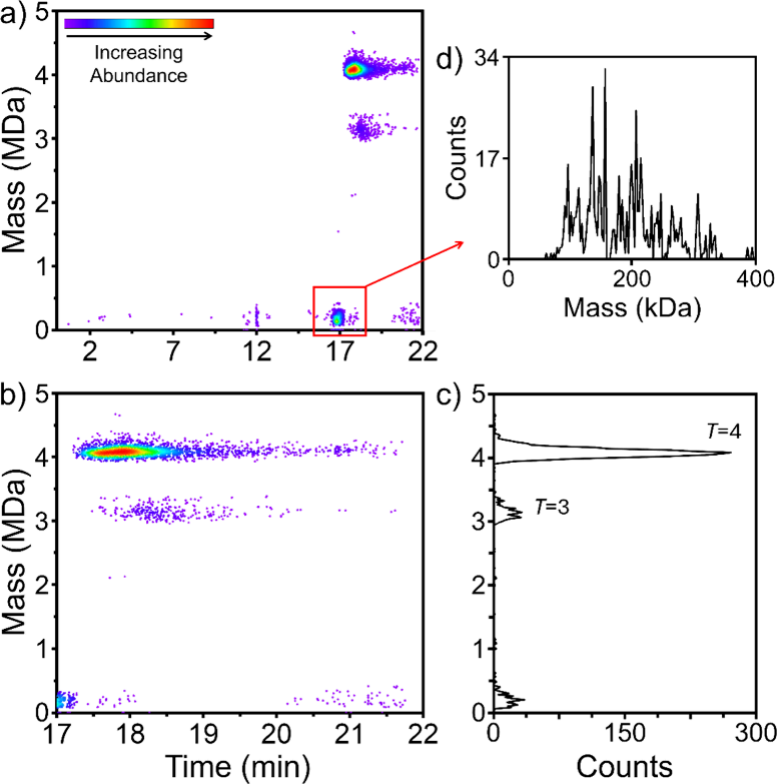
SEC-CD-MS analysis of HBV CP149 VLPs. (a) 2D abundance map showing the full elution time range from injection to 22 min. (b) 2D abundance map showing an expanded view of the 17 to 22 min elution time range. (c) CD-MS mass distribution showing the mass range from 0 to 5 MDa. (d) Expanded view of the CD-MS mass distribution for the low mass feature in (a) at an elution time of 17 min.

The HBV capsid is polymorphic, assembling from CP dimers to form *T* = 3 and *T* = 4 icosahedra with 90 and 120 CP dimers, respectively.^40,41^*In vivo* around 5% of the capsids are *T* = 3.^42^*In vitro*, the HBV truncated core protein CP149 (which lacks the C-terminal nucleic acid binding domain) assembles into *T* = 3 and *T* = 4 VLPs.^43^ A representative 2D abundance map for the SEC-CD-MS analysis of an HBV CP149 assembly reaction is shown in Figure 4a. An expanded view of the 17–22 min elution time range is shown in Figure 4b and the mass distribution of the eluting ions is shown in Figure 4c. There are three main features, the *T* = 4 VLP which has a mass of around 4 MDa and elutes at around 17.8 min, the *T* = 3 VLP which has a mass of around 3 MDa and elutes at around 18.4 min, and a much lower mass feature which elutes at around 16.9 min. An expanded view of the CD-MS mass distribution for the lower mass feature is shown in Figure 4c. The masses range from around 80 kDa up to around 340 kDa. There are a number of sharp peaks in the mass distribution which appear to be regularly spaced. However, the spacing between the peaks (around 20.5 kDa) does not match the mass of a capsid protein dimer (33 541 Da) and so they must be due to impurities. Their short elution time suggests they have large hydrodynamic radii which, for masses around 200 kDa, indicates a solution structure that is not compact (for example, a random coil). This feature is discussed further below.

As noted above, free HBV dimer persists in solution with the VLPs. This free dimer is responsible for the second peak in the UV SEC chromatograms for HBV in Figure 1. However, the free dimer was not detected in the SEC-CD-MS measurements in Figure 4 because the electrospray source was turned off before it elutes.

The expected masses for the *T* = 3 and *T* = 4 capsids are 3.019 MDa and 4.025 MDa, respectively. The measured masses (from Figure 4c) are 3.110 and 4.059 MDa. Other SEC-CD-MS runs gave slightly different values, but they are all significantly larger than the expected masses, and the excess mass for the *T* = 3 is consistently larger than for the *T* = 4.

The masses of large complexes electrosprayed from volatile salt solutions (native MS) are usually slightly larger than the expected mass because of salt adducts. While low molecular weight salts are easily separated from the larger analytes by SEC, some salts remain stubbornly attached to large analytes and are not removed by SEC. In previous CD-MS studies, the excess mass for HBV CP149 *T* = 3 and *T* = 4 VLPs was around 0.3%.^44^ However, in these studies, 250 nm electrospray emitters were used with flow rates <0.2 μL/min. The larger deviations observed here probably result from the high flow rate from the SEC column, and the use of 15 μm electrospray emitters to accommodate the high flow rate. The high flow rate leads to larger electrospray droplets and more salt adducts. The excess mass could be reduced by decreasing the flow to the electrospray emitter or by introducing other methods to reduce adducts after the ions enter the CD-MS instrument.

The larger excess mass for the *T* = 3 noted above may be because a species with around 90 dimers is an intermediate in the assembly and disassembly of the *T* = 4 particle.^45,46^ This species has a mass similar to the *T* = 3 particle (which has exactly 90 dimers) but it is thought to have a different, lower symmetry, structure and hence it is has a more heterogeneous mass distribution.

### Orthogonal VLP Characterization Using SEC-MALS

SEC-MALS has been widely used to characterize both biological macromolecules and bionanoparticles, often with additional online dynamic light scattering (DLS), UV, and RI detectors.^47,48^ The signals from these nondestructive online detectors help identify peaks of interest by simultaneously measuring several biophysical properties. These properties include molar mass, radius of gyration (*R*_g_, when greater than 10 nm), hydrodynamic radius (*R*_h_), conformation, UV extinction coefficient, concentration, and payload for protein conjugates or viral capsids. Since SEC-MALS can be used to determine these biophysical quantities data slice by data slice (0.5 s for this study), it provides information on peak heterogeneity but lacks the resolution of CD-MS.

**Figure 5:**
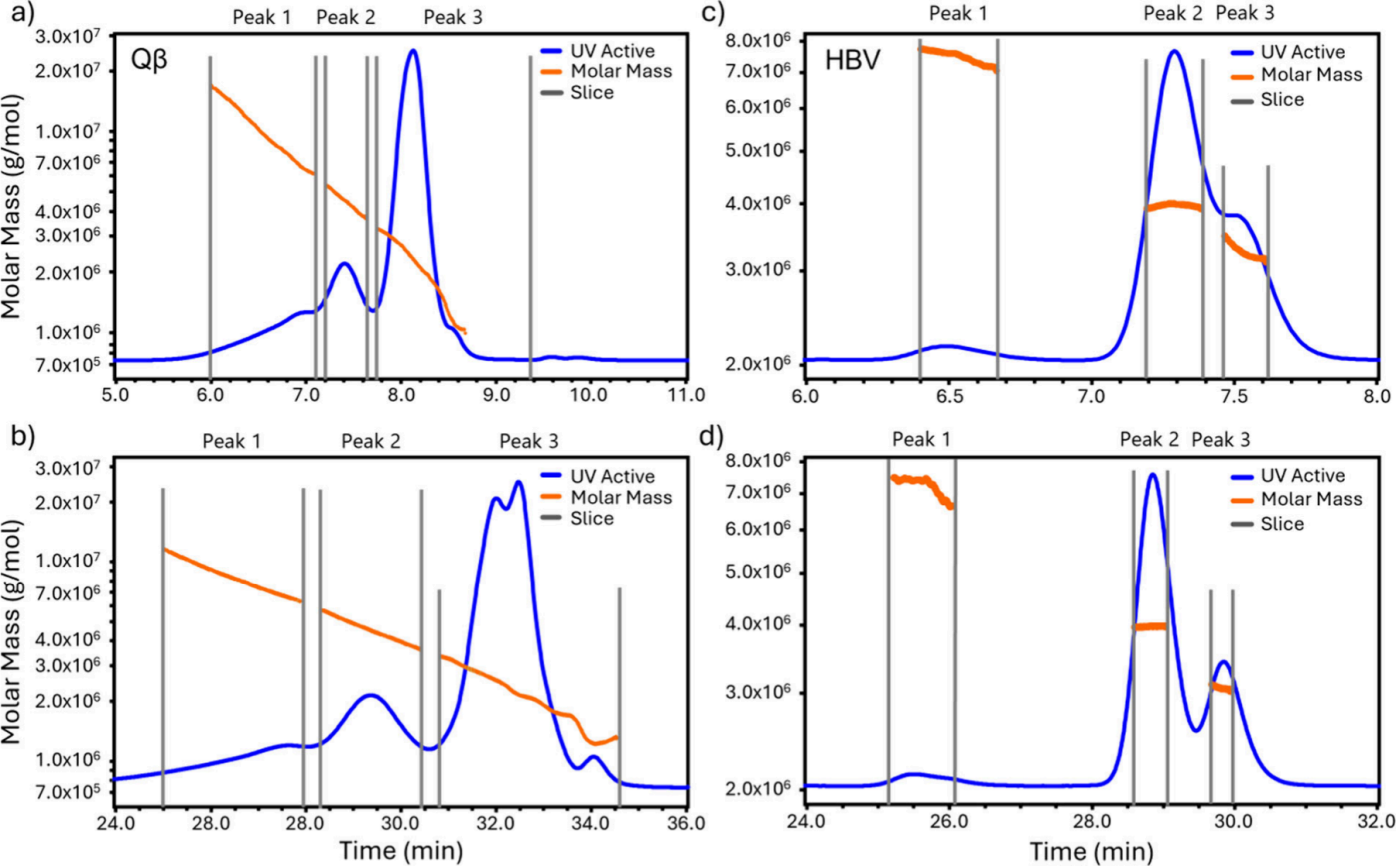
SEC-MALS results for Qβ and HBV VLPs. Molar mass versus elution time (orange points) and UV chromatogram measured at 280 nm (blue line) for Qβ at flow rates of (a) 1.15 mL/min and (b) 0.29 mL/min, and the same for HBV at (c) 1.15 mL/min and (d) 0.29 mL/min.

Both Qβ and HBV VLPs were analyzed by SEC-MALS using a wide bore column (7.8 mm ID) at flow rates of 0.29 and 1.15 mL/min. The molar masses for Qβ across the eluting peaks are plotted against time in Figure 5a and b (orange line). The UV chromatogram measured at 280 nm is overlaid (blue line). The SEC resolution is better at the lower flow rate. The molar masses from SEC-MALS at the two flow rates are tabulated alongside those from CD-MS in Table S3 in Supporting Information.

Qβ peak 3 spans a wide elution time range (from 30.5 to 34.5 min at 0.29 mL/min and from 7.7 to 8.7 min at 1.15 mL/min), with molar masses from 1.0 to 3.3 MDa. This range of molar masses encompasses the range of distinct masses observed by CD-MS and hence peak 3 in Figure 5 is assigned to the geometric variants identified by CD-MS (see Figure 3b). The Qβ variants are only partially resolved by the SEC, resulting in the steady decrease in the molar mass across the eluting peaks. The small partially resolved peak at an elution time of 34 min is attributed to the *T* = 1 particle at 1.09 MDa in the CD-MS measurements (Figure 3b), while the other CD-MS peaks (at 1.67, 2.03, 2.59, and 3.0 MDa) are attributed to the broader, more intense peak centered on 32.25 min. The peak at 1.09 MDa is the largest peak in the CD-MS spectrum (Figure 3b) where it accounts for 44% of the ions with masses between 1 MDa and 3.5 MDa. The peak attributed to the *T* = 1 particle in the SEC-UV results is much smaller. However, UV absorption and CD-MS measure different quantities. UV absorption measures the mass density (in mg/mL, for example), while CD-MS measures the number density. To convert the CD-MS number density measurements to mass density, the number of particles in each mass bin should be multiplied by their mass, enhancing the high mass features. Figure S5 shows a comparison of the CD-MS number density distribution for Qβ to the mass density distribution obtained by multiplying the number of particles by their mass.

Peaks 1 and 2 of the Qβ VLP sample have weight-average molar masses around 4x and 2x the molar mass of peak 3, respectively. These peaks are attributed to Qβ aggregates which were detected at an integrated intensity of around 19% of the total. For CD-MS the integrated intensity of the aggregates was around 5%. However, when mass weighted to account for the difference in the measurements described above, the fraction of multimers in the CD-MS measurements increases to 18%, which is in good agreement with the aggregate fraction detected by SEC-UV. Note, however, that two aggregate components were detected by SEC-UV while the aggregate mass distribution detected by SEC-CD-MS showed a single broad component with peaks (see Figure S2c for CD-MS mass weighted distribution). The average mass of the aggregates in the CD-MS mass weighted distribution is 5.5 MDa.

Figure 5c and d shows the SEC-MALS results for the HBV VLP sample. The figure shows molar masses for HBV VLPs determined by MALS across the eluting peaks (orange line) with the chromatogram determined by UV absorption overlaid (blue line). Results are shown for flow rates of 1.15 mL/min then at 0.29 mL/min. The results with the lower flow rate again have better resolution (Figure 5d). In contrast to the Qβ VLP results, the molar masses across the HBV VLP peaks are much more uniform, due to the lower heterogeneity of the HBV VLP sample. The molar masses from SEC-MALS at the two flow rates are tabulated alongside those from CD-MS in Table S4 in Supporting Information. The molar masses of peaks 2 and 3 are close to the expected values for *T* = 4 and *T* = 3 capsids, respectively. The biophysical characterization of Qβ and HBV VLPs by SEC-MALS is described in Supporting Information.

HBV peak 1 has molar masses around 7 MDa, close to the expected mass for HBV VLP dimers. However, HBV VLP dimers were not observed in the SEC-CD-MS measurements.

Peak 1 is small, only around 2% of the total intensity. However, such a minor component should be detected easily by CD-MS. This is not a problem with the detection of high mass multimers by CD-MS: multimers for other samples (e.g., AAV) are routinely detected.^49^ Inspection of the SEC-CD-MS results for HBV VLPs in Figure 4 shows that there is a component with a shorter elution time than the peak due to the HBV VLPs. However, this component has a broad distribution of masses extending from under 100 kDa to over 300 kDa, not the 7 MDa masses found by SEC-MALS. The most likely explanation for these seemingly conflicting results is that the 7 MDa species observed in the SEC-MALS measurements is an aggregate of the 100–300 kDa species, the aggregate is not stable when transferred into the gas phase, and it dissociates into small fragments. If this is true then the 7 MDa species observed in the SEC-MALS measurements is not an HBV VLP dimer, but an aggregate of much smaller species.

## Conclusions

In the past, the slow data acquisition rate of single ion MS methods has hindered its direct coupling to chromatography without compromising mass resolution. A few years ago, it would typically take 30–60 min to measure a CD-MS spectrum of 5000 ions with good mass resolution. With recent advances in high throughput CD-MS, the same spectrum can now be measured in 30–60 s *without discrimination or distortion of the mass distribution*. This progress, and the use of narrow bore columns facilitates the direct coupling of SEC to CD-MS, bringing the precision and accuracy of MS methods to separations of heterogeneous and high mass samples that cannot be analyzed by conventional MS.

Adding together results from multiple injections provides another way to increase signal intensity and circumvent the time scale mismatch between chromatography and single ion MS. However, the use of multiple injections leads to longer measurement times and consumes more sample. Recently, Marty and co-workers showed that Hadamard transforms can be used to reduce the measurement time associated with multiple injections,^50^ however, this approach still consumes more sample, and it is more technically challenging than a single injection. As we show here, with high throughput CD-MS, enough ions can be collected with a single injection. Marty and co-workers used Orbitrap DMT and the measurements were limited to samples with molar masses less than a megadalton.

In this study, we have coupled SEC to high throughput CD-MS and performed measurements into the megadalton regime. This work brings high precision mass measurements to SEC separations of high mass analytes for the first time. We have demonstrated the resolution of protein complexes, antibodies, and two VLP samples. The technology developed here is extendable to a broad range of high mass analytes, including nanoparticles, vaccines, and gene therapies, into the gigadalton regime. In addition to the separation and identification of analytes, SEC-CD-MS facilitates the identification of low abundance features like aggregates, and it can be used to distinguish stable long-lived solution aggregates from weakly bound aggregates and electrospray artifacts. The coupling of SEC to CD-MS opens the door to the direct coupling of CD-MS to other chromatography methods like reverse phase LC and affinity chromatography. Finally, CD-MS and SEC-MALS complement each other. Combining the high-resolution and mass accuracy of CD-MS with the biophysical characterization achieved by SEC-MALS provides a more in-depth characterization of large macromolecules which are becoming increasingly prevalent in the biopharmaceutical industry. A sophisticated and powerful toolbox is essential for effectively analyzing these complex macromolecules, ensuring reliable and comprehensive results that drive innovation and progress in the field.

## Supporting Information Available

Materials and samples; SEC-CD-MS mobile phase ramp; Qβ CD-MS masses and assignments; SEC-CD-MS results for BSA; SEC-CD-MS results for Qβ aggregates; SEC-CD-MS separation of Qβ VLP and thyroglobulin; SEC-CD-MS serial dilution for Qβ; SEC-MALS and SEC-CD-MS results for Qβ; number density and mass density distributions for Qβ; SEC-MALS and SEC-CD-MS results for HBV VLPs; biophysical characterization of Qβ and HBV VLPs by SEC-MALS (PDF)
