# Supplementary material for: Coupling of Size Exclusion Chromatography to High Throughput Charge Detection Mass Spectrometry for the Analysis of Large Proteins and Virus-like Particles
Source: Anal Chem. 2025 Jan 29;97(5):3036–44. doi: 10.1021/acs.analchem.4c06084 (PMC11822739; doi:10.1021/acs.analchem.4c06084)
Supplement: Supplementary file 1 — ac4c06084_si_001.pdf [file ac4c06084_si_001.pdf]

Supporting Information

Coupling of Size Exclusion Chromatography to High Throughput Charge Detection Mass Spectrometry for the Analysis of Large Proteins and Virus Like Particles

Raj A Parikh,[1] Lohra M Miller,[2] Benjamin E Draper,[2] Lavelay Kizekai,[3] Balasubrahmanyam Addepalli,[3] Michelle Chen,[4] Matthew A Lauber,[3]* Martin F Jarrold[1]*

[1] *Chemistry Department, Indiana University, Bloomington, Indiana 47405 USA*
[2] *Megadalton Solutions Inc, 3750 E Bluebird Ln, Bloomington, Indiana 47401 USA*
[3] *Waters Technology Corporation, 34 Maple Street, Milford, MA, 01757, USA*
[4] *Waters Technology Corporation, 6330 Hollister Ave, Goleta, CA 93117 USA*
Corresponding authors' email addresses: matthew_lauber@waters.com
and mfj@iu.edu

Table of Contents

Materials and Samples

Mobile phase ramp used for LC-CD-MS

Masses and tentative assignments of the five Qβ CD-MS mass peaks

Expanded view of SEC-CD-MS results for BSA

Ion current chromatogram for Qβ showing aggregates and mass distribution of the aggregates

SEC-CD-MS separation of a Qβ VLP and thyroglobulin

SEC-CD-MS serial dilution study for bacteriophage Qβ VLP

Comparison of SEC-MALS and SEC-CD-MS results for Qβ VLP

Comparison of number density and mass density distributions for Qβ

Comparison of SEC-MALS and SEC-CD-MS results for HBV VLPs

Biophysical characterization of Qβ and HBV VLPs by SEC-MALS.

Materials and Samples

Ammonium acetate (AmAc) (09691), OmniSolv LC-MS water (WX0001), bovine thyroglobulin (T1001), and bovine serum albumin (BSA) (A2153) were purchased from Millipore Sigma. Formic acid was purchased from VWR (64-18-6). Bacteriophage Qβ VLPs were purchased from Fina Biosolutions. NIST monoclonal reference standard 8671 (NISTmAb) was purchased from National Institute of Standards and Technology Office of Reference Material (Lot 14HB-D-002) and truncated hepatitis B virus capsid protein (CP149) (kindly provided by Prof. Adam Zlotnick of Indiana University) was assembled in 300 mM sodium chloride for 24 h to yield T=3 and T=4 icosahedral VLPs with 90 and 120 capsid protein dimers, respectively. Details of the assembly protocol can be found elsewhere. The HBV VLP assembly reaction is strongly concentration dependent with a pseudo critical CP concentration beyond which all additional CP goes into VLP formation. The pseudo critical concentration of the HBV CP dimer (typically 3-5 μM depending on the conditions) is always present in solutions that contain VLPs. After assembly, the HBV VLPs and residual CP149 dimer concentration were 0.67 mg/mL.

Table S1 Mobile phase ramp used for SEC-CD-MS separations.

**Time (min)**	**Flow Rate (μl/min)**	**Solvent A (water + 0.1% formic acid)**	**Solvent B (200 mM ammonium acetate)**
0.0	35	1	99
20.0	35	1	99
20.5	20	1	99
30.0	10	100	0

Table S2 Masses and tentative assignments of the five Qβ CD-MS mass peaks.

**Peak**	1	2	3	4	5
**Mass (MDa)**	1.09	1.67	2.03	2.59	3.0
**Number of CPs**	60	90	120	150	180
**Structure**	T=1	T=1 (prolate)	T=2	T=3 (oblate)	T=3

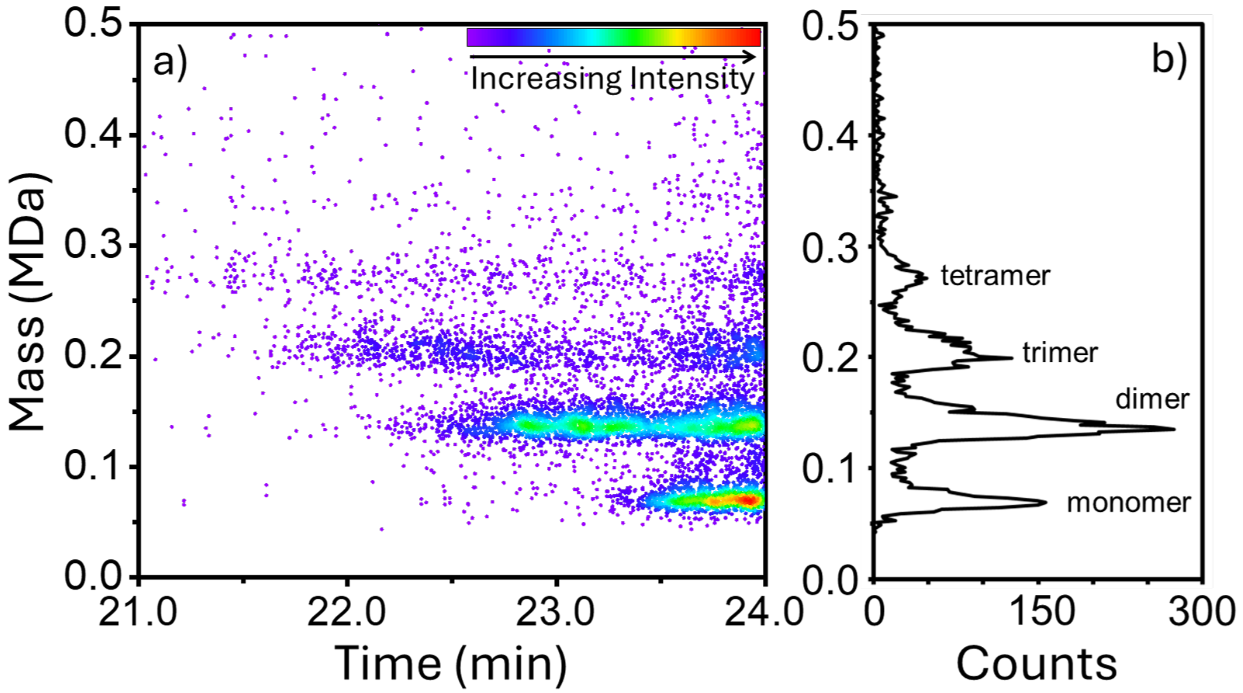

Figure S1 Expanded view of SEC-CD-MS results for BSA. a) 2D abundance map and b) mass distribution. Features due to the monomer, dimer, trimer and tetramer of BSA are evident in both the 2D abundance map and the mass distribution. For the monomer the intensity occurs between 23.4 min and the cut-off at 24 min. For the dimer and trimer there is intensity at the same retention time as the monomer. This intensity results from short-lived, weakly-bound dimer or trimer and/or dimer and trimer generated in the electrospray process. There is also intensity at shorter times that results from long-lived, strongly bound dimers and trimers present in solution.

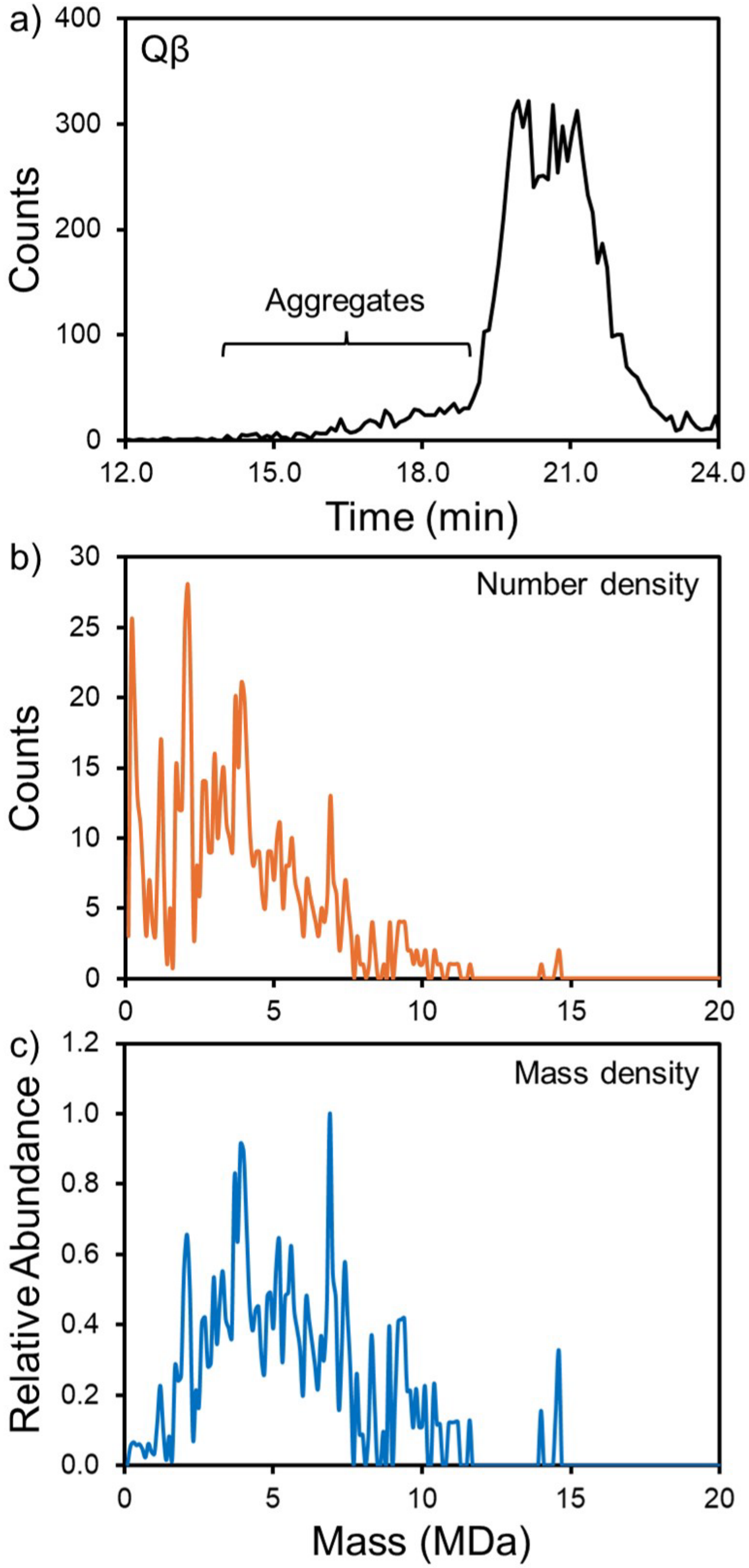

Figure S2 Aggregates of Qβ by SEC-CD-MS. a) Total ion current chromatogram for Qβ showing intensity at shorter elution times (13-19 min) that is attributed to aggregates. b) CD-MS mass distribution for ions with elution times between 13- and 19-min. c) Mass distribution in b) converted from number density to mass density for comparison with UV-SEC.

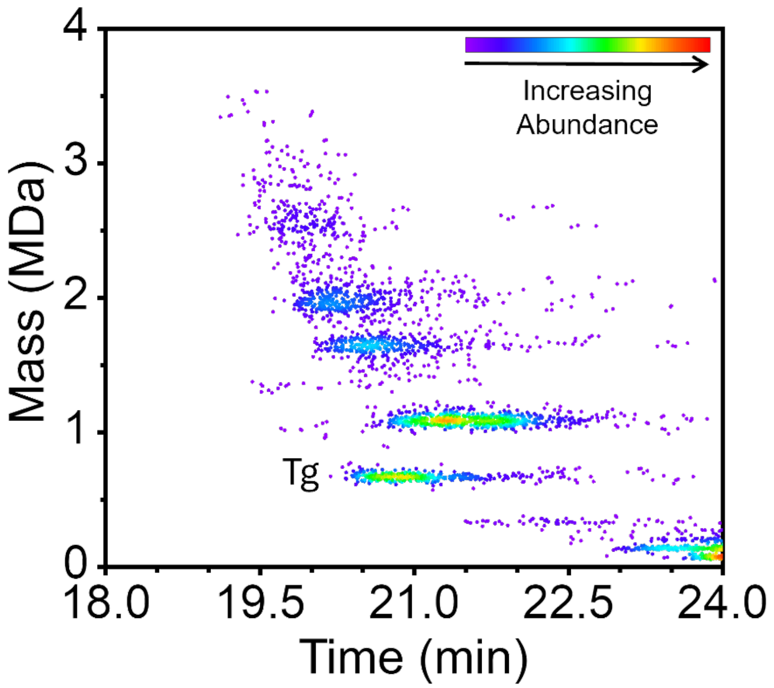

Figure S3 2D abundance map of the SEC-CD-MS separation of a Qβ VLP and thyroglobulin mixture using BSA to mark the end of the elution. The Tg label shows the main feature due to the thyroglobulin dimer. Most of the other features in the 2D abundance map are due to Qβ, and they have the same masses and retention times as the Qβ features in Figure 3 in the main text. The thyroglobulin dimer elutes at 20.75 min as evident in Figure 2. Because of the large number of components that coelute between 19.75 min and 22.00 min, a UV detector would be unable to provide useful information about this sample. The large number of overlapping and unresolved m/z peaks present in the m/z spectrum rules out conventional MS. However, with the mass resolution provided by CD-MS, it is possible to identify species and determine their elution times, even when they are not completely resolved in the chromatogram.

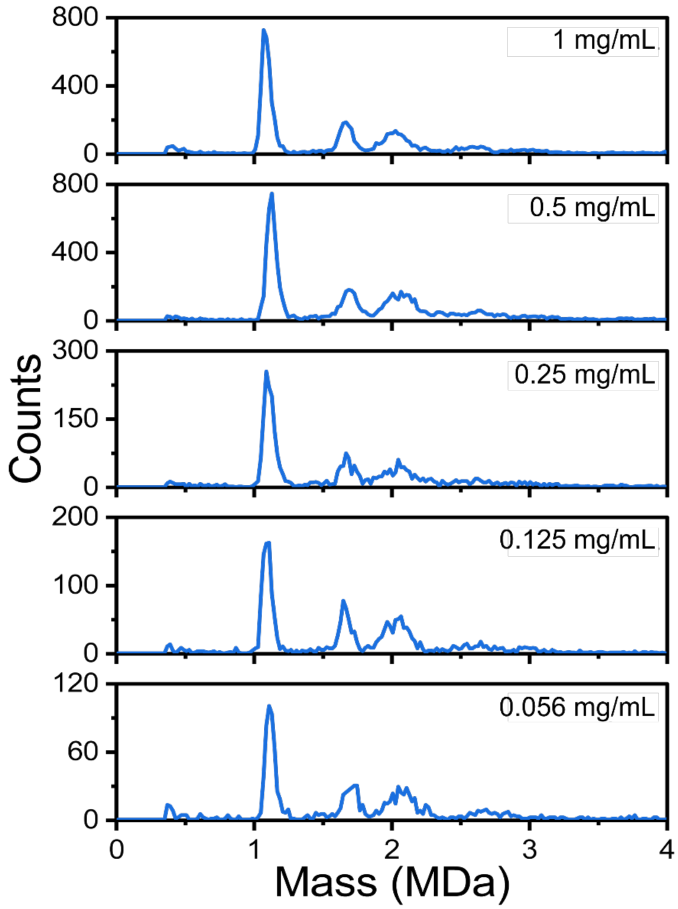

Figure S4 SEC-CD-MS serial dilution study for bacteriophage Qβ VLP. Mass distributions recorded for concentrations ranging from 1.0 mg/mL to 0.056 mg/mL.

Table S3 Tabulated SEC-MALS and SEC-CD-MS results for Qβ VLP.

	Peak 1		Peak 2		Peak 3		
	SEC-MALS	CD-MS	SEC-MALS	CD-MS	SEC-MALS		CD-MS
Flow Rate (mL/min)	Mw (MDa)	Mw (MDa)	Mw (MDa)	Mw (MDa)	Mw Range (MDa)	Mw (MDa)	Mw (MDa)
0.29	7.89	5.5 MDa	4.51	5.5 MDa	1.23-3.34	2.27	1.09, 1.67, 2.03, 2.59, 3.0
1.15	8.45		4.44		1.03-3.22	2.21	

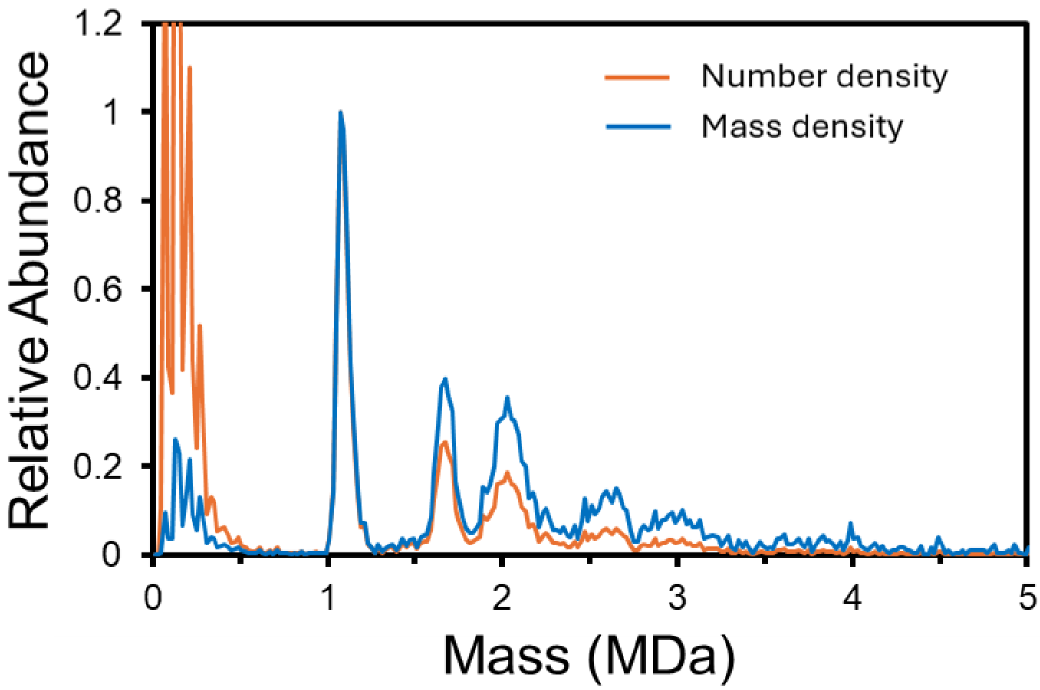

Figure S5 Comparison of number density distribution measured by CD-MS for Qβ to mass density distribution obtained by multiplying the number of particles at each mass by their mass. The mass density distribution is measured using UV-SEC.

Table S4 Tabulated SEC-MALS and SEC-CD-MS results for HBV VLPs.

	Peak 1		Peak 2		Peak 3	
	SEC-MALS	CD-MS	SEC-MALS	CD-MS	SEC-MALS	CD-MS
Flow Rate (mL/min)	Mw (MDa)	Mw (MDa)	Mw (MDa)	Mw (MDa)	Mw (MDa)	Mw (MDa)
0.29	7.23	N/D	3.97	4.06 MDa	3.07	3.11
1.15	7.50		3.95		3.25	

Biophysical characterization of Qβ and HBV VLPs by SEC-MALS.

Qβ and HBV VLPs, which have the same elution time, have the same hydrodynamic radius as measured by the online DLS detector. This is consistent with the expectation for an ideal SEC separation. However, the molar masses for these two samples are different. In addition, the main species for these two VLPs have similar Rg/Rh ratios of approximately 0.86, suggesting a spherical structure. Combining the molar mass and radii results, we can conclude that the non-aggregated structures of the two VLP samples have similar shapes but different densities. Using the online UV and RI signals, we also calculated an average extinction coefficient value of 3.2 and 2.05 mL/(cm.mg) for Qβ and HBV VLPs, respectively. These additional biophysical parameters revealed by the online detectors following SEC, combined with the resolution and accurate mass capability of CD-MS, enables a full characterization of complex VLP samples.
